# Self-enforcing regional vaccination agreements

**DOI:** 10.1098/rsif.2015.0907

**Published:** 2016-01

**Authors:** Petra Klepac, Itamar Megiddo, Bryan T. Grenfell, Ramanan Laxminarayan

**Affiliations:** 1 Department of Applied Mathematics and Theoretical Physics, University of Cambridge, Cambridge, UK; 2 Center for Disease Dynamics, Economics and Policy, Washington, DC 20036, USA; 3 Ecology and Evolutionary Biology, Princeton University, Princeton, NJ 08544, USA; 4 Woodrow Wilson School of Public and International Affairs, Princeton University, Princeton, NJ 08544, USA; 5 Fogarty International Center, National Institutes of Health, Bethesda, MD 20892, USA; 6 Public Health Foundation of India, New Delhi 110070, India

**Keywords:** transboundary movement, regional cooperation, epidemic dynamics, SIR model, metapopulation

## Abstract

In a highly interconnected world, immunizing infections are a transboundary problem, and their control and elimination require international cooperation and coordination. In the absence of a global or regional body that can impose a universal vaccination strategy, each individual country sets its own strategy. Mobility of populations across borders can promote free-riding, because a country can benefit from the vaccination efforts of its neighbours, which can result in vaccination coverage lower than the global optimum. Here we explore whether voluntary coalitions that reward countries that join by cooperatively increasing vaccination coverage can solve this problem. We use dynamic epidemiological models embedded in a game-theoretic framework in order to identify conditions in which coalitions are self-enforcing and therefore stable, and thus successful at promoting a cooperative vaccination strategy. We find that countries can achieve significantly greater vaccination coverage at a lower cost by forming coalitions than when acting independently, provided a coalition has the tools to deter free-riding. Furthermore, when economically or epidemiologically asymmetric countries form coalitions, realized coverage is regionally more consistent than in the absence of coalitions.

## 1. Background

Infectious diseases are a transnational problem that cannot be solved by countries acting unilaterally. Because infections easily spread from one country to another, controlling infectious diseases regionally requires international cooperation and coordination of efforts. The need for cooperation in control infectious diseases was recognized as early as the 1850s, when advances in transportation and ease of travelling facilitated the spread of cholera epidemics across Europe and to North America [1]. However, cooperation has not yet been formally included in the modelling framework used to design immunization strategies. The World Health Organization issues important guidelines and recommendations, but compliance with those guidelines is voluntary, and control strategies are usually set and implemented by countries independently. By focusing on strongly immunizing vaccine-preventable diseases, here we use a coupled economic and epidemiological model to explore factors that can motivate coalition formation and promote cooperation among countries in designing and implementing regional immunization strategies.

The performance of a vaccination campaign depends on transmission rates, classically framed in terms of the basic reproduction ratio, *R*_0_, or the expected number of new cases caused by a single infected case in an immunologically naive population [2]. Paediatric mass vaccination at a level *p* against an immunizing infection reduces *R*_0_ to an effective value, *R*_E_ *=* *R*_0_(1 − *p*), which leads to a well-known threshold for herd immunity, *p*_c_ *=* 1 − 1*/R*_0_ [2]. The underlying model involves a homogeneous, well-mixed population, but the qualitative prediction is robust: immunizing above the herd immunity threshold, *p_c_*, leads to the local elimination of transmission and the prevention of disease [2,3].

In order to decide on the best strategy, it is necessary to take economic as well as epidemiological factors into consideration. Vaccination programmes are costly, and when these costs are explicitly balanced against the benefits of reduced transmission and fewer cases, the best vaccination strategy can lie anywhere from no intervention to local elimination, depending on the relative costs of vaccination and infection [4]; relatively non-pathogenic infections with expensive vaccines may generate an economic optimum vaccination rate below the elimination level. While only four diseases are targeted for global elimination (polio, guinea worm, malaria and yaws), many more are controlled by routine vaccination (e.g. rubella, mumps, rotavirus diarrhoea, *Haemophilus influenzae* type b, pertussis, diphtheria, tetanus, meningococcus C and pneumococcus) that primarily provides early protection against infections that are most dangerous for the very young. Childhood immunizations prevent 2.5 million deaths per year and have the potential to save another two million deaths each year, mostly children under the age of five [5]. Here, we focus on strongly immunizing vaccine-preventable diseases that are not necessarily aimed for global elimination, and explore whether a cooperative regional vaccination approach can improve national vaccination strategies.

To address the question of cooperation in a formal setting, we model a region that aims to optimize its vaccination strategy against a strongly immunizing infection. We assume that vaccination needs to continue indefinitely even in the case of local elimination to protect against imported infections or to prevent a related pathogen to take advantage of the niche vacated by elimination [6]. National vaccination strategies reflect local interests, socioeconomic conditions and public health priorities, and, as a result, can vary greatly within a region. Disease dynamics in different countries of the region are linked by cross-border movement of infected individuals, and depend on the strength of population interchanges between them. Countries with low vaccination coverage can therefore act as a source of infection to their neighbours.

We allow countries to coordinate a regional vaccination strategy by formation of coalitions through international agreements and apply it to the control of infectious diseases and the nonlinear dynamics that govern their spread. We find that by forming coalitions and deciding on a joint vaccination strategy, countries can achieve higher vaccination coverage at a lower cost than when acting independently, and that under certain conditions, a cooperative strategy of this kind is stable. This result opens the way to the more efficient use of existing public health resources.

## 2. Self-enforcing international agreements

Many environmental problems, such as depletion of the ozone layer, pollution of air and the oceans, and climate change, have a feature in common with infectious diseases, which is that they are transboundary or global in nature and countries cannot solve them by acting alone. The theory of international environmental agreements (IEAs) offers useful insights for studying transnational public goods, such as the protection of the Earth's ozone layer, greenhouse gas emissions reduction, climate change mitigation and water management [7,8]. To reach a common goal, countries form coalitions, but there is no international body that can enforce these agreements. The theory of IEAs tells us when such coalitions succeed even though compliance is voluntary.

IEAs can be modelled in a game-theoretic framework where countries first decide independently whether or not to join the coalition, and then quantify their joint environmental goals (e.g. pollution abatements) either simultaneously [7] or with signatories taking the lead [8]. Because coalition members' abatement choice is an increasing function of the number of member countries, the coalition implicitly employs a carrot-and-stick mechanism: when a country joins, the coalition rewards it by increasing abatement, and if it leaves, then the coalition punishes it (and itself) by reducing it. At the equilibrium, there is no incentive to leave (known as *internal stability*) or join the coalition (known as *external stability*)—the coalition is stable or self-enforcing [7–9].

Among identical countries, stable coalitions are rare and agreements signed by all countries are unlikely owing to free-riding [10]. If the difference between the global net benefits under non-cooperative (countries acting independently) and fully cooperative outcomes is large, so is the incentive to free-ride, and the self-enforcing IEA cannot support a large number of countries [8]. To increase participation and stability, coalition can employ a number of measures, such as penalizing free-riding [8], offering transfers [7,10,11], imposing trade sanctions [10,12] or linking environmental protection to other international agreements, such as those facilitating technology transfers [10,13].

## 3. Methods

We adapt the theory of IEAs to the particular problem of transnational epidemiological dynamics. We incorporate self-enforcing agreements in a metapopulation model for dynamics of infectious diseases and consider their application for design and implementation of regional control strategies for immunizing infections. We consider only strongly immunizing infections and vaccines that mimic this immunity. Rapidly evolving pathogens such as influenza require a more complex framework allowing for different strain dynamics, host history of infection and immunity [14,15] and are therefore not further considered here.

Coalitions are added to an explicit spatial SIR model where *n* populations are coupled through migration of infected individuals (following [16,17])3.1
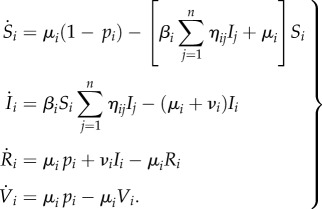


Here, *S_i_*, *I_i_* and *R_i_* are respective proportions of susceptible, infected and recovered individuals in population *i*, births are balancing deaths at the rate *μ_i_* and the infection on average lasts 1/*ν_i_*. A proportion *p_i_* of the individuals are vaccinated at birth (at the end of maternal immunity). Different populations are coupled through movement of infected individuals, captured with the coupling matrix *η*, given by3.2
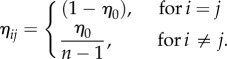


The amount of transboundary movement across different numbers of coupled countries (*n*) is symmetric to conserve population sizes. This coupling reflects short trips made by individuals, rather than permanent migration or relocation of individuals.

For each population *i*, we distinguish between costs of vaccination *c*(*p_i_*), that capture immunization programmes' implementation and operation costs and increase exponentially with the proportional increase in vaccination coverage (as supported by data, e.g. see figure 4 in [18]), and infection costs *c_Ii_* that capture direct and indirect costs of disease (e.g. morbidity, mortality and loss of productivity) and so are proportional to the equilibrium prevalence of infection [4],3.3
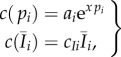
with the total cost3.4
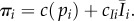


The cost of setting up a vaccination campaign in location *i* is *a_i_* and the increase in vaccination cost for high coverage is captured by *x* (chosen to reflect that achieving 80% coverage costs about five times as much to achieve 20% coverage).

We first consider countries that are identical in their parameters for transmission rate, costs of vaccination and infection. In the second part of the paper, we consider the interaction of asymmetric countries to capture the heterogeneity in countries' epidemiological and economic conditions.

### 3.1. Self-enforcing vaccination agreements

Drawing on the theory of IEAs [8,11,19], we introduce self-enforcing agreements to the management and control of immunizing infections. Initially, we model coalition formation for symmetric countries.

We set up a two-stage game. In the first stage, countries decide whether or not to join a coalition. In the second stage, the set of *k* countries comprising the coalition chooses their vaccination coverage 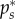 that minimizes the combined costs of vaccination and disease burden of the coalition (*π_C_* = *k*π*_s_* because countries are identical)3.5
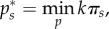
while incorporating the non-signatories' reaction function in their cost minimization. Countries outside of the coalition (non-signatories) then minimize their local costs *π*_ns_ independently,3.6
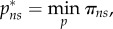


### 3.2. Stability

A coalition of *k* countries is self-enforcing or stable if no member has the incentive to leave (internally stable), and no non-member has the incentive to join (externally stable). A coalition is internally stable if the local cost of each member country (*π*_*s*_) is lower in the *status quo* than its cost should it leave the coalition, 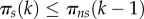. A coalition is externally stable if each non-member's local cost is lower than its cost should it choose to accede, 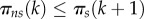. By definition, *k* = 0 is internally and *k* = *n* is externally stable [7–9].

### 3.3. Travel restrictions

To increase cooperation and deter free-riding and defecting, the coalition can impose a *travel restriction* by limiting movement of infected individuals across its borders. This can be achieved, for example, by reducing overall travel, by requiring proof of vaccination or by introducing border surveillance systems to detect symptomatic individuals. Travel restriction acts here as a punishment (like trade sanctions in [10,12]). Sanctions have a cost for signatories as well as for non-signatories, and the coalition will implement them only when their benefits outweigh their costs.

We then look at the effects of sanctions on the stability of coalitions and on willingness to accede to a coalition. Following [20], we assume that the coupling parameter *η* can be reduced by imposing travel restrictions that limit cross boundary movement of infected between signatories and non-signatories. We let *Q* be the total number of direct and indirect costs involved in travel restrictions and assume that *Q* affects the coupling parameter between signatories (*s*) and non-signatories (*ns*) so that
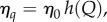
such that3.7
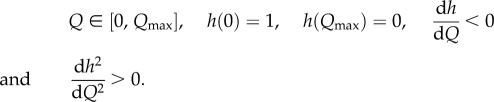
3.8
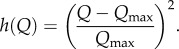


When there are no restrictions imposed, the coupling parameter *η_q_* is equal to *η*_0_. Full intensity of travel restrictions results in complete isolation (there is no coupling), preventing any cross-border movement. Both signatories and non-signatories incur the direct and indirect cost of travel restrictions (for example, direct costs by implementing the restrictions and indirect ones through loss of trade),3.9
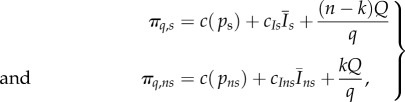
where *q* is a scaling parameter. For the coalition size *k* and the cost of travel restrictions *Q* between any two countries, the total cost incurred by the coalition is (*n* − *k*)*Q* and the sum of the costs incurred by non-members is *kQ*.

In the second stage of the game, in addition to choosing the vaccination level countries simultaneously choose the intensity of travel restrictions. The coalition optimal strategy is the combination of vaccination coverage (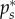) and travel restriction intensity (*h*(Q*)) that minimize the joint coalition costs 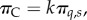 given the non-signatories choice.3.10
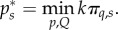


### 3.4. Heterogeneity

As the cost of implementing travel restrictions can be prohibitive, we next consider ways to promote coalition participation in the absence of restrictions. While we first considered a metapopulation of identical countries, we now include regional heterogeneity by allowing epidemiological and economic parameters to vary between countries (equation (3.1)).

When countries are asymmetric, there are 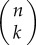 ways tomake a coalition of size *k* among the total of *n* countries, and 2*^n^* − (*n* + 1) possible coalitions in total (i.e. 1 < *k* ≤ *n*). To minimize its combined costs of vaccination and infection in the coalition *C* (*π*_C_), the coalition now optimizes the vaccination level for each of the countries in coalition 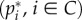 resulting in a vector 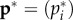 of optimal strategies3.11
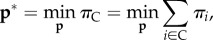
where *π_i_* is given by equation (3.4).

In this case, coalitions of the same size can experience different optimal levels of vaccination coverage and associated prevalence and costs, depending on which countries are inside (or outside) the coalition. Furthermore, in coalitions of size *k*, country *i* can have different optimal vaccination strategies 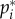 depending on cost and epidemic parameter values of other members and non-members. The range of optimal outcomes 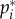 is illustrated using summary statistics showing the mean values and fifth and 95th quantiles.

### 3.5. Numerical simulations

All simulations were coded using the MATLAB programming language version R2012a and its Optimization Toolbox and performed on Princeton University's Adroit computing cluster (eight node Beowulf cluster). Equilibrium values are determined using the fsolve function, which finds a root of the nonlinear system of equations with the equilibrium value of the non-coupled system as the initial condition for the solver. The minimization of different cost functions is performed using the nonlinear programming solvers fminbnd and fmincon, which respectively find a minimum of constrained nonlinear single-variable (for a local optimum of a single country) and multivariate functions (for a global optimum of the system of *n* countries). The minimization procedure is constrained over the interval 0 ≤ *p_i_* ≤ 1, where 1 < *i* < *n* and is subject to adjoint equations of the model described by equations (3.1) and (3.2). For the model with travel restrictions, additional constraints are given by equation (3.7). Simulations of the model with heterogeneity find regional and local optima as described above for all the countries over all 2*^n^* − *n* possibilities (the non-cooperative outcome and all the possible coalitions).

## 4. Results

To study the effect of coalitions on vaccination coverage, we refine a basic two-patch SIR model for immunizing infections that includes economic constraints [4] in two significant ways. First, we explore a system of *n* countries coupled through transboundary movement of infected individuals. Second, we combine the game theory of international agreements with the dynamic epidemiological model to allow relatively complex patterns of coalitions in vaccine deployment (Methods). Our analysis first focuses on a set of identical countries (equal epidemiological and economic parameters).

**Figure 1.**
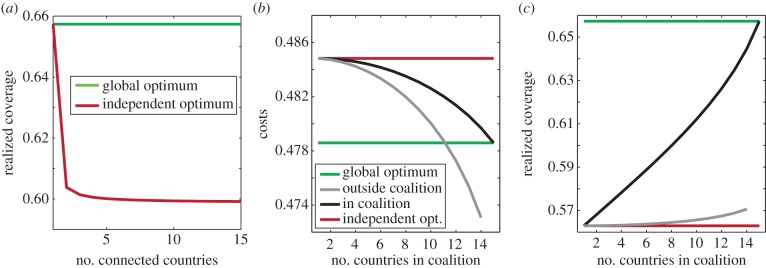
Multi-patch SIR model. (*a*) Global, fully cooperative optimum (green line) and the independent, non-cooperative optimum (Nash equilibrium), given in red for increasing numbers of identical interconnected countries. Parameters: *R*_0_ = 5, coupling strength = 10*μ*/(*n* − 1), *a_i_* = 0.1, *c_Ii_* = 5. Costs (*b*) and realized coverage (*c*) for a system of 15 identical interconnected countries, for increasing coalition size (*x*-axis). Green and red lines show global and independent optima (as in (*a*)), black lines show the optimum realized by the countries in the coalition, grey lines show optimum for countries that have not joined the coalition. Other parameters as in (*a*).

If countries act independently, the result is the Nash equilibrium [7,8]; each one chooses a vaccination strategy that minimizes its local costs, and no country can profit by unilaterally changing its strategy (local optimum—figure 1, red line). Full cooperation is achieved if all countries try to minimize their combined costs, or if a global planner can enforce a cost-minimizing policy (global optimum—figure 1, green line). For highly coupled regions, the independent, non-cooperative optimum results in lower vaccination coverage (but at a higher cost) than the fully cooperative outcome described by the global optimum [4]. Increasing the coupling or the number of interconnected countries increases this mismatch between global (figure 1*a*, green line and electronic supplementary material, figure S1) and independent optima (figure 1 and electronic supplementary material, figure S1 red line). The realized coverage in each country is lower, but the sustained cost is higher (figure 1*b,c* and electronic supplementary material, figure S1). Note that for the set of parameters used in this example (and assuming we cannot stop immunizing), the global optimum is not elimination—the coverage is below the herd immunity threshold, and the disease prevalence is above zero.

### 4.1. Coalitions

The optimal outcome for the members of coalitions is to increase their vaccination coverage compared with the case when countries act independently. As a result of high coverage in the coalition (figure 1*c*, black line), non-signatories can experience fewer incoming infections and may experience lower costs compared with non-cooperative outcome (when no countries form coalition). The non-signatories select a level of vaccination coverage that minimizes their individual costs (figure 1*b,c* grey line). Higher coverage in the coalition reduces the prevalence and infection-related costs, making further resources available for vaccination. The coverage in the coalition depends on the number of signatories: when a country joins, the coalition's vaccination coverage increases (figure 1*c*, black line).

### 4.2. Stability

**Figure 2.**
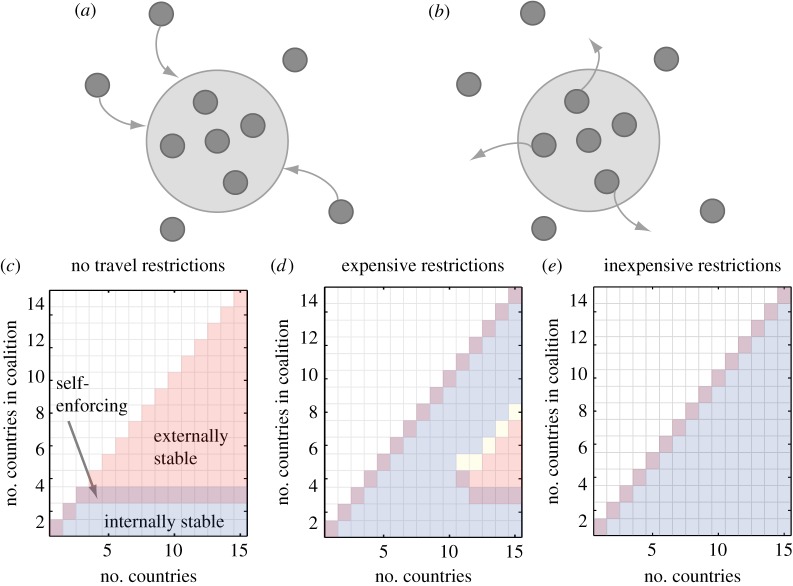
Coalition stability. (*a*) Externally unstable coalition—benefits of the coalition are greater than the free-riding pay-off, giving non-signatories the incentive to join. (*b*) Internally unstable coalition—benefits of free-riding are greater than benefits from coalition giving signatories an incentive to defect. (*c–e*) Effects of travel restrictions on coalition stability for different numbers of interconnected countries *n* (*x*-axis) and for increasing numbers of signatories *k* (*y*-axis). Blue shading shows internally stable coalitions, externally stable coalitions are shaded orange, and their overlap shows self-enforcing coalitions. Coalitions in yellow are neither externally nor internally stable, and area in white shows unfeasible coalitions. *R*_0_ = 5, coupling strength = 20*μ*/(*n* − 1), *a_i_* = 0.1, *c_Ii_* = 5. (*c*) No travel restrictions, (*d*) expensive travel restrictions, *q* = 1000, (*e*) inexpensive travel restrictions, *q* = 5000.

**Figure 3.**
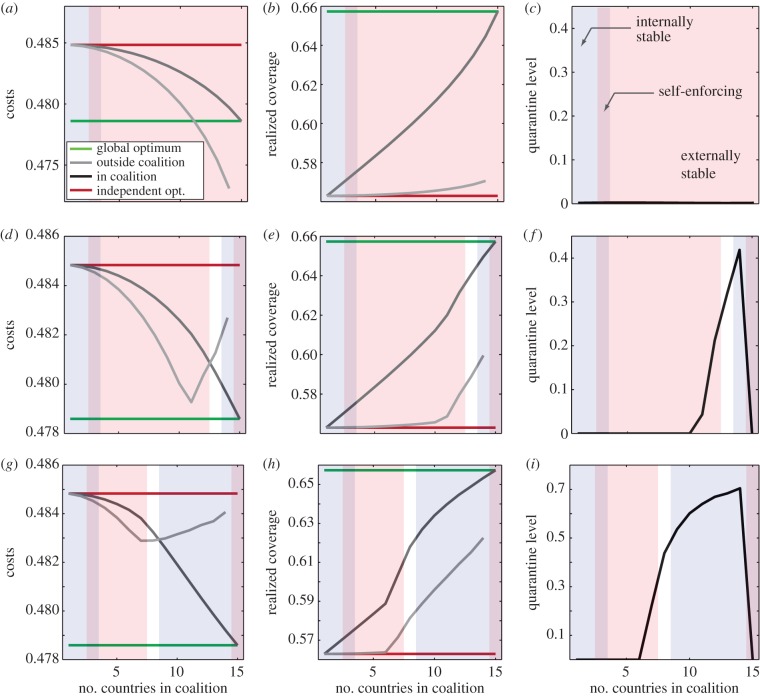
Multi-patch SIR model with 15 coupled countries. Costs (*a,d,g*), realized coverage (*b,e,h*) and intensity of travel restrictions (*c,f,i*) for three control scenarios: no travel restrictions (*a–c*); expensive travel restrictions, *q* *=* 500 (*d–f*); inexpensive travel restrictions, *q* *=* 1000 (*g–i*). Green lines show vaccination coverage at the global optimum (*b*,*e*,*h*) and the corresponding costs (*a*,*d*,*g*). Red lines show realized coverage and corresponding costs when countries are acting independently (Nash equilibrium). Black and grey lines show optimal coverage and corresponding costs for signatories and non-signatories, respectively. Internally stable coalitions are shaded blue, externally stable coalitions are shaded orange, and their overlap shows self-enforcing coalitions. Coalitions in white are unstable. Parameters: *R*_0_ = 5, coupling strength = 20*μ*/(*n* − 1), *a_i_* = 0.1, *c_Ii_* = 5.

Stability of a coalition depends on countries' costs in the coalition versus outside the coalition (see schematics in figure 2*a,b* for unstable coalitions). Signatories to small coalitions have a lower cost inside the coalition than what their cost would be should they leave the coalition; these coalitions are internally stable (figure 2*c–e* and figure 3*a–c* blue shading, *k* ≤ 3). For larger coalitions that achieve high coverage, non-signatories benefit from the avoided incoming infections, and therefore they have a lower cost by acting independently than if they joined the coalition. In this case, there is no incentive to join: the coalition is externally stable (figure 2*c–e* and figure 3*a–c* orange shading*, k* ≥ 3). The larger the coalition, the higher immunization costs become relative to infection costs, increasing incentives to free-ride. At the equilibrium, countries have no incentive either to leave or to join the coalition: the coalition is stable (figure 2*c–e* and figure 3*a–c* purple shading, *k* = 3 in this case). In stable coalitions, countries voluntarily adhere to the regional strategy and cooperatively increase their coverage: agreements are self-enforcing. Similar to the solution in environmental agreements [7,8], the self-enforcing vaccination agreement cannot support a large number of identical countries.

### 4.3. Travel restrictions

To prevent disease spread, policymakers can implement control at borders, require proof of vaccination or impose travel restrictions. Travel restrictions have direct costs for both non-signatories and for the coalition (electronic supplementary material, figures S2*g* and S3*g* grey line), but they benefit the coalition in two ways. First, they directly reduce the number of infections imported into signatory countries. Second, travel restrictions isolate non-signatories, stopping them from free-riding on elevated immunization levels in the coalition; a cost for non-signatories (compare grey lines in figure 3*a,d,g*). The combination of isolation and lack of benefits from free-riding, leads to a shift in costs that deters free-riding, and non-signatories are incentivized to expand their immunization coverage (figures S2*e* and S3*e* in electronic supplementary material, grey line; note the increase in non-signatories' cost of coverage in the presence of travel restrictions), resulting in a higher vaccination coverage in the entire region. With limited free-riding on its efforts, the coalition signatories also increase their own immunization levels (figures S2*e* and S3*e* in electronic supplementary material). Both signatories and non-signatories incur costs of travel restrictions according to their relative sizes, so travel restrictions are costly for small coalitions and for individual non-signatories, relative to large coalitions.

In our simulations with *n* *=* 15 countries, when travel restrictions are expensive to implement, other than the grand coalition where *k* = *n*, only coalitions of size *k* ≥ 10 choose to use the strategy (figure 3*f* and electronic supplementary material, figure S2*d,g*). When travel restrictions are inexpensive, excluding the grand coalition, coalitions of size *k* ≥ 6 choose to implement them (figure 3*i* and electronic supplementary material, figure S3*d,g*). For coalition members, the benefits of reduced disease burden from protection by travel restrictions (electronic supplementary material figures S2*f* and S3*f*, black line) outweigh the direct costs of implementing restrictions (compare black lines in figures S2*f,g* and S3*f,g* in electronic supplementary material). The restriction is therefore a credible threat.

Because of the resulting change in costs, the payoffs are larger inside the coalition, and fully cooperative coalition (*k* = *n*) becomes stable or self-enforcing (purple shading figure 2*d,e*, figure 3*d–i*, see also electronic supplementary material, figures S2 and S3). Note that the travel restrictions are not implemented in any of the stable coalitions—it is the credible threat of restrictions that encourages both joining and remaining in the coalition.

### 4.4. Heterogeneity

**Figure 4.**
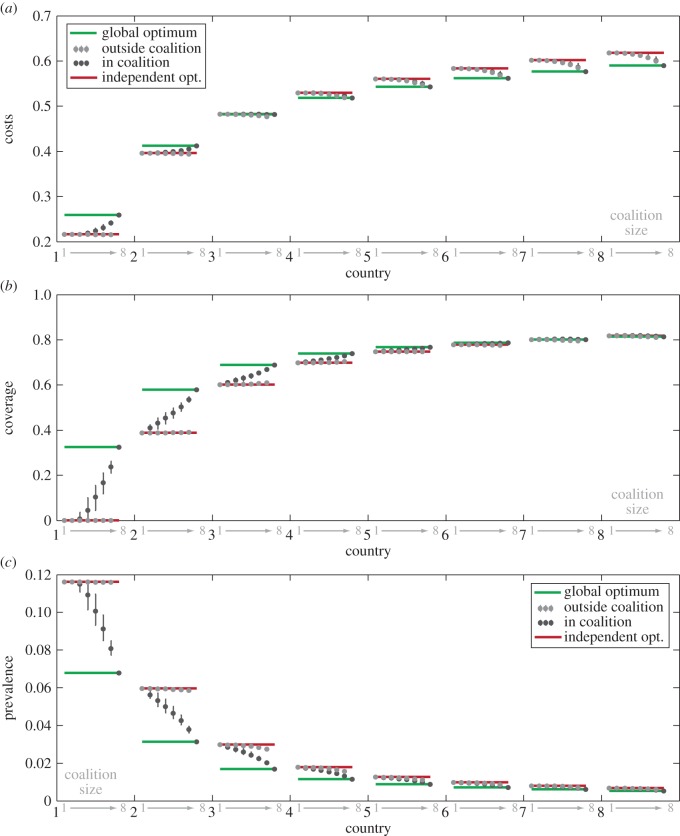
SIR model for a system of eight asymmetric interconnected countries showing summary statistics for costs (*a*), coverage (*b*) or prevalence (*c*). For each country (shown on *x*-axis in black), the coalition sizes (indicated in grey on *x*-axis) are ordered from 1 (non-cooperative outcome) to 8 (fully cooperative outcome). There are 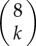 possible coalitions of size *k*, and here we show the mean value and range of optimization outcomes for a given country in a coalition of a given size. Circles show mean costs (*a*), coverage (*b*) or prevalence (*c*) for each country and each coalition size when that country is in coalition (black) and outside of coalition (grey). Whiskers show fifth and 95th quantiles. Red and green lines show independent and global optimum for each country, respectively. Cost of infection parameter varies linearly across countries from *c_I_*_1_ = 1 for country 1, and *c_I_*_8_ = 15 for country 8. *R*_0_ = 5, coupling strength = 10*μ*/(*n* − 1), *a_i_* = 0.1. All 248 optimizations are shown in electronic supplementary material, figure S4.

Finally, we account for the spatial heterogeneity in costs, disease burden or resources between countries in a model without travel restrictions. We show the results for a metapopulation of eight countries where cost of infection varies linearly across countries from *c_I_*_1_ = 1 for country 1, and *c_I_*_8_ = 15 for country 8 in figure 4 (see also electronic supplementary material, figure S4). In this case, *R*_0_ is the same for all countries (*R*_0_ = 5, *p_c_* = 0.8). The difference in cost parameters leads to a range of local optimal vaccination levels for different countries in a non-cooperative setting (red lines in figure 4*b* and electronic supplementary material, figure S4). Heterogeneities in costs of vaccination or *R*_0_ lead to qualitatively similar results (see electronic supplementary material, figures S5 and S6). With the heterogeneity in costs of infection, local optima range from no vaccination for country 1, to vaccinating above the elimination threshold *p_c_* for country 8 (red line in figure 4*b* ranges from 0 to greater than 0.8). A country with low vaccination coverage can now act as a source of infections for its well-vaccinated neighbour. Even though vaccination coverage in country 8 is above the elimination threshold *p*_c_, its prevalence is above zero (figure 4*c*); it cannot reach elimination because of incoming infections from other countries.

With eight countries, there are 247 possible coalitions of *k* ≥ 2 and one non-cooperative outcome, in which none of the countries form a coalition (see electronic supplementary material, figure S4 for an overview of all of the 248 optimizations). Resulting optima for a given country can vary greatly in different coalitions of the same size, depending on the parameter values of other countries inside and outside of the coalition (electronic supplementary material, figure S4). For example, there are 35 coalitions of four countries with country 1 as a member, and in those coalitions, the optimal coverage for country 1 varies from 0% to 12% (electronic supplementary material, figure S4). Figure 4 summarizes these results with circles showing the mean optimal values for a given country and a coalition of a given size, and whiskers showing the fifth and 95th quantiles. For each country, we plot its summary statistics for increasing coalition sizes—coalition size of 1 shows the non-cooperative outcome (also given by the red line) and coalition size 8 shows the fully cooperative outcome (also given by the green line).

**Figure 5.**
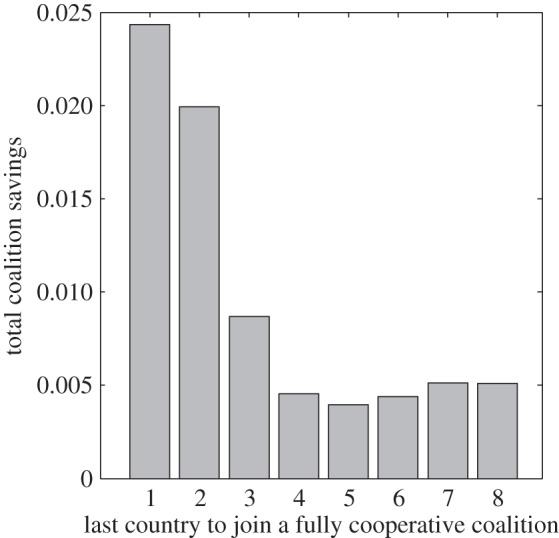
Overall coalition savings when a coalition becomes a fully cooperative coalition (all countries participate, *k* = *n*), with a country that joins the coalition last indicated on the *x*-axis. Parameter values as in figure 4.

In a fully cooperative outcome (figure 4 green lines), optimal vaccine coverage is considerably higher particularly for the countries with previously low coverage (e.g. compare red and green lines in figure 4 for country 1: vaccination coverage increases from 0 to greater than 30%). The cost of elevating coverage for these countries is high, but the savings of their neighbours through avoided infections more than compensate for these costs. As more countries join the coalition, the vaccination coverage among signatories increases (figure 4*b*, black circles) and approaches the global optimum (figure 4*b*, green lines). Coverage among non-signatories (figure 4*b* grey dots) remains comparable to the non-cooperative outcome (figure 4*b* red lines) although they enjoy slightly lowered costs owing to free-riding (figure 4*a*, grey dots). Compared with the non-cooperative outcome (figure 4*b*, red lines), differences in coverage levels and prevalence decrease as the coalition approaches full cooperation (figure 4*b,c*, note the decreased range of green compared with than red lines; see also electronic supplementary material, figure S6 where reductions in prevalence are achieved at very little cost). Overall, the fully cooperative coalition achieves higher vaccination coverage at a lower cost than smaller coalitions, with some countries benefiting more than others (figure 4*a* and see also electronic supplementary material, figure S4). Countries with low perceived cost of infection (countries 1 and 2 in figure 4*a*) experience an increase in their costs compared with non-cooperative outcome. If overall costs of the coalition decrease when it becomes fully cooperative (all countries are members), then its members can promote participation by compensating the countries that would otherwise incur an increase in costs. The coalition can become fully cooperative in eight different ways—each respective country can be the last one to join. Because of asymmetries in parameter values, the costs incurred when increasing the size of the coalition from seven to eight members will differ in each of these scenarios. Regardless of which country joins the coalition last the overall benefit of the coalition is positive (figure 5), even though some countries can incur a cost from joining a coalition (electronic supplementary material, figure S7). With heterogeneity, the differences in incurred or perceived costs between countries have the potential to be used as compensation to increase coalition participation, leading to elevated and more consistent vaccination coverage in the region.

Heterogeneities in costs or epidemic parameters can be exploited in terms of redistribution of benefits obtained by increased vaccination coverage to stabilize the fully cooperative coalition even without the threat of sanctions. A more comprehensive analysis of asymmetries is needed to fully identify where and how transfer systems can be used to increase global welfare in infectious diseases prevention.

### 4.5. Caveats and future directions

The epidemic model is deliberately kept simple in this initial study, but is subject to important caveats. Vaccines often induce immunity that wanes over time (e.g. pertussis [21]) or can provide strong selection pressure that allows for emergence and spread of viral immune escape variants [22–24]. Demographic parameters such as birth rate can greatly influence and drive the epidemic dynamics [25–27], whereas age structure affects the spread of the disease and case fatality patterns [28,29], suggesting that infection costs should also vary with age. Stochastic effects, amplified by seasonality in transmission, can lead to fade-outs at lower levels of coverage than predicted by deterministic models [30–32].

Here we model dynamics of infectious diseases and vaccination strategies on a population level. While individuals' vaccine-seeking behaviour and response to interventions contributes to the outcome and success of public health campaigns, incorporating this behaviour in mechanistic models of disease dynamics can be challenging [33] and is not further considered here.

Finally, we look only at interactions between countries. Donors, non-governmental organizations and foundations play very important roles in the global health arena (e.g. Rotary International and the Bill & Melinda Gates Foundation in polio eradication and the Carter Center in the case of neglected tropical diseases). Their incentives and interventions, together with the political and economic setting in which control strategies are framed, will be a contributing determinant of the success of coordinated control efforts.

## 5. Conclusion

The extensive literature on the spread of infectious diseases [2,3] and the economics of their control by vaccination [34–36] has not addressed the question of when regional coalitions for regional disease control through vaccination form and under what conditions they are stable. The theory of IEAs [12] offers a useful baseline on coalition formation and here we extend it and apply it to infectious diseases and their nonlinear dynamics.

We study international coordination of immunization efforts by linking self-enforcing coalitions with epidemiological dynamics in a game-theoretic setting where countries are coupled by transnational movement of infections. The effectiveness of a coalition and the attractiveness of free-riding are also a function of coupling, or the interconnectedness of populations. As the transboundary coupling in this model represents short trips made by the individuals, and not permanent migration, the strength of coupling is expected to be high and is set to 10 or 20 times the population turnover in our analysis. International transportation data show that coupling rates are very heterogeneous and region-specific, and the values we consider are well within the range observed in data. In 2005, there were more than 440 million international tourist arrivals in Europe [37] compared with nine million births [38], whereas Africa during the same period had 37 million international arrivals [39] and around 30 million births [40].

When identical countries are coupled through the transboundary movement of infections, global health benefits are substantially higher in the fully cooperative outcome than when countries act independently. That cooperative outcomes are better than non-cooperative ones is considered conventional wisdom, especially in economic literature [8,10,41–43]), but this wisdom has not fully percolated to other fields. By cooperating in the efforts to control infectious diseases by means of immunization, we find that countries can achieve much higher vaccination coverage at a lower cost than when acting independently. This suggests that coalitions may be helpful in improving the use of existing resources and open up the funding for increasing vaccination coverage or for other public health issues. However, because of incentives to free-ride, large coalitions cannot be sustained in a self-enforcing manner in the absence of sanctions (as it is widely reported in economic literature [8,10,41–43]).

Sanctions are commonly used in IEAs to increase coalition participation and to ensure that signatories are meeting the goals [10,12]. In the case of vaccination agreements, nonlinearities in infectious disease dynamics provide a trade-off between investing in the population immunity and the prevalence of infection in the population. With a threat of travel restrictions, instead of free-riding on the high coverage in the coalition, the non-signatories invest in higher local immunity to avoid high costs of infection. As a result, non-signatories realize higher vaccination coverage in the presence than in the absence of imposed restrictions. The threat of sanctions in this case leads to a substantial increase in vaccination coverage both inside and outside the coalition.

Travel restrictions have been used to control the spread of SARS [44] and Ebola virus [45,46], but their effectiveness is limited [47,48] especially for diseases with presymptomatic transmission like influenza [49–51]. Furthermore, travel restrictions are considered controversial owing to their adverse economic impact [52,53] and prohibitive cost of implementation. Epstein *et al.* [54] estimate that extensive restrictions would cost the US 0.8% of its GDP, amounting to over $130 billion based on 2013 data [55]. We therefore also consider factors that could stabilize coalitions in the absence of any restrictions.

Countries in a region can vary greatly in their epidemic, demographic or socioeconomic characteristics. This existing heterogeneity can be used to promote coalition participation even in the absence of hard-to-implement restrictions. Heterogeneity in cost and epidemiological parameters results in diverse local vaccination optima and asymmetric countries experience varying levels of savings (or costs) by joining the coalition. This heterogeneity can increase coalition participation when countries that benefit from the coalition compensate others to join and increase their vaccination coverage. As disparities in realized coverage among countries decrease inside the coalition, the vaccination coverage in the region becomes not only higher, but also more consistent.

While many countries are adopting policies for universal coverage of childhood vaccines, vaccination coverage is far from universal in many locations and increasing coverage in those places will require more investment. There are particular disparities in local vaccination coverage in the case of measles in sub-Saharan Africa [56], especially in remote areas [57], making this system a good candidate for policy interventions that foster coalitions. Another example where a coalition approach would be useful is in funding immunization campaigns with the new meningococcal meningitis conjugate vaccine in the 25 countries of the African meningitis belt [58–60]. In those examples, marginal benefit from vaccine uptake in a neighbouring country can be significant enough to warrant regional agreements.

Local infectious disease dynamics are one reason why coalitions are so effective in increasing vaccination coverage. Outbreaks, once sparked, depend predominantly on the local effective transmission rate (*R*_E_ > 1)—i.e. the build-up of local infectives [61]. Whereas importations of disease depend on the herd immunity achieved by vaccination (supply of the good by all countries) and the strength of connectivity with other regions, the size of a local outbreak depends on the number of non-immune individuals. If everybody in the population is immunized, then the importations will not lead to additional infections.

Most environmental issues are dynamically different. For example, all countries contribute to the atmospheric accumulation and mixing of ozone-depleting substances, such as chlorofluorocarbons (CFCs). If only one country stops its CFCs emissions, then the thickness of the ozone layer directly above it will not improve; ozone layer protection requires long-term commitment from nearly all countries in the world. On the other hand, immunizing infections are dominated by local nonlinearities arising from herd immunity. Regional and global coordination is necessary to regulate disease importations and coordinate control efforts. In addition, vaccines not only directly protect people who have been vaccinated, but also provide indirect protection to those unvaccinated by reducing overall transmission. Local nonlinear dynamics of infectious diseases that unfold over short periods and the indirect protection of vaccines make the control of immunizing infections particularly fitting for a regional approach.

## Supplementary Material

Supporting Material for “Self-enforcing regional vaccination agreements”

## References

[1] Huber V 2006 The unification of the globe by disease? The International Sanitary Conferences on cholera, 1851–1894. Hist. J. 49, 453 (10.1017/S0018246X06005280)

[2] Anderson RM, May RM 1991 Infectious diseases of humans. Oxford, UK: Oxford University Press.

[3] Keeling MJ, Rohani P 2008 Modeling infectious diseases in humans and animals. Princeton, NJ: Princeton University Press.

[4] Klepac P, Laxminarayan R, Grenfell BT 2011 Synthesizing epidemiological and economic optima for control of immunizing infections. Proc. Natl Acad. Sci. USA 108, 14 366–14 370. (10.1073/pnas.1101694108) PMC3161560 21825129

[5] Levine OS, Bloom DE, Cherian T, de Quadros C, Sow S, Wecker J, Duclos P, Greenwood B 2011 The future of immunisation policy, implementation, and financing. Lancet 378, 439–448. (10.1016/S0140-6736(11)60406-6) 21664676

[6] Lloyd-Smith JO 2013 Vacated niches, competitive release and the community ecology of pathogen eradication. Phil. Trans. R. Soc. B 368, 20120150 (10.1098/rstb.2012.0150) 23798698 PMC3720048

[7] Carraro C, Siniscalco D 1993 Strategies for the international protection of the environment. J. Public Econ. 52, 309–328. (10.1016/0047-2727(93)90037-T)

[8] Barrett S 1994 Self-enforcing international environmental agreements. Oxford Econ. Pap. 46, 878–894.

[9] d'Aspremont C, Jacquemin A, Gabszewicz JJ, Weymark JA 1983 On the stability of collusive price leadership. Can. J. Econ. 17–25. (10.2307/134972)

[10] Carraro C, Siniscalco D 1998 International environmental agreements: incentives and political economy. Eur. Econ. Rev. 42, 561–572. (10.1016/S0014-2921(97)00118-9)

[11] Barrett S 2001 International cooperation for sale. Eur. Econ. Rev. 45, 1835–1850. (10.1016/S0014-2921(01)00082-4)

[12] Barrett S 1997 The strategy of trade sanctions in international environmental agreements. Resour. Energy Econ. 19, 345–361. (10.1016/S0928-7655(97)00016-X)

[13] Wagner UJ 2001 The design of stable international environmental agreements: economic theory and political economy. J. Econ. Surv. 15, 377–411. (10.1111/1467-6419.00143)

[14] Francis T 1960 On the doctrine of original antigenic sin. Proc. Am. Philos. Soc. 104, 572–578.

[15] Kucharski A, Gog JR 2012 Influenza emergence in the face of evolutionary constraints. Proc. R. Soc. B 279, 645–652. (10.1098/rspb.2011.1168) PMC3248728 21775331

[16] Grenfell BT, Bolker BM, Kleczkowski A 1995 Seasonality and extinction in chaotic metapopulations. Proc. Royal Soc. Med. 259, 97–103. (10.1098/rspb.1995.0015)

[17] Keeling M, Rohani P 2002 Estimating spatial coupling in epidemiological systems: a mechanistic approach. Ecol. Lett. 5, 20–29. (10.1046/j.1461-0248.2002.00268.x)

[18] Freuling CM, Hampson K, Selhorst T, Schröder R, Meslin FX, Mettenleiter TC, Müller T 2013 The elimination of fox rabies from Europe: determinants of success and lessons for the future. Phil. Trans. R. Soc. B 368, 20120142 (10.1098/rstb.2012.0142) 23798690 PMC3720040

[19] Telser LG 1980 A theory of self-enforcing agreements. J. Bus. 53, 27–44. (10.1086/296069)

[20] Rowthorn R, Laxminarayan R, Gilligan C 2009 Optimal control of epidemics in metapopulations. J. R. Soc Interface 6, 1135–1144. (10.1098/rsif.2008.0402) 19324686 PMC2817149

[21] Lavine JS, King AA, Bjornstad ON 2011 Natural immune boosting in pertussis dynamics and the potential for long-term vaccine failure. Proc. Natl Acad. Sci. USA 108, 7259–7264. (10.1073/pnas.1014394108) 21422281 PMC3084147

[22] Restif O, Grenfell BT 2007 Vaccination and the dynamics of immune evasion. J. R. Soc. Interface 4, 143–153. (10.1098/rsif.2006.0167) 17210532 PMC2358969

[23] Park AW, Daly JM, Lewis NS, Smith DJ, Wood JLN, Grenfell BT 2009 Quantifying the impact of immune escape on transmission dynamics of influenza. Science 326, 726–728. (10.1126/science.1175980) 19900931 PMC3800096

[24] Pepin KM, Volkov I, Banavar JR, Wilke CO, Grenfell BT 2010 Phenotypic differences in viral immune escape explained by linking within-host dynamics to host-population immunity. J. Theor. Biol. 265, 501–510. (10.1016/j.jtbi.2010.05.036) 20570681 PMC4537168

[25] Grenfell B, Bjornstad O, Finkenstadt B 2002 Dynamics of measles epidemics: scaling noise, determinism, and predictability with the TSIR model. Ecol. Monogr. 72, 185–202. (10.1890/0012-9615(2002)072%5B0185:DOMESN%5D2.0.CO;2)

[26] Pitzer VE et al. 2009 Demographic variability, vaccination, and the spatiotemporal dynamics of rotavirus epidemics. Science 325, 290–294. (10.1126/science.1172330) 19608910 PMC3010406

[27] Bonds MH, Rohani P 2010 Herd immunity acquired indirectly from interactions between the ecology of infectious diseases, demography and economics. J. R. Soc. Interface 7, 541–547. (10.1098/rsif.2009.0281) 19740924 PMC2842798

[28] Andreasen V 1989 Disease regulation of age-structured host populations. Theor. Popul. Biol. 36, 214–239. (10.1016/0040-5809(89)90031-2) 2814905

[29] Metcalf CJE, Lessler J, Klepac P, Cutts F, Grenfell BT 2012 Impact of birth rate, seasonality and transmission rate on minimum levels of coverage needed for rubella vaccination. Epidemiol. Infect. 140, 2290–2301. (10.1017/S0950268812000131) 22335852 PMC3487482

[30] Bolker B, Grenfell B 1996 Impact of vaccination on the spatial correlation and persistence of measles dynamics. Proc. Natl Acad. Sci. USA 93, 12 648–12 653. (10.1073/pnas.93.22.12648) PMC38047 8901637

[31] Ferrari MJ, Grais RF, Bharti N, Conlan AJK, Bjornstad ON, Wolfson LJ, Guerin PJ, Djibo A, Grenfell BT 2008 The dynamics of measles in sub-Saharan Africa. Nature 451, 679–684. (10.1038/nature06509) 18256664

[32] Ferrari MJ, Grenfell BT, Strebel PM 2013 Think globally, act locally: the role of local demographics and vaccination coverage in the dynamic response of measles infection to control. Phil. Trans. R. Soc. B 368, 20120141 (10.1098/rstb.2012.0141) 23798689 PMC3720039

[33] Funk S, Bansal S, Bauch CT, Eames KTD, Edmunds WJ, Galvani AP, Klepac P 2015 Nine challenges in incorporating the dynamics of behaviour in infectious diseases models. Epidemics 10, 21–25. (10.1016/j.epidem.2014.09.005) 25843377

[34] Geoffard P-Y, Philipson T 1997 Disease eradication: private versus public vaccination. Am. Econ. Rev. 87, 222–230.

[35] Bauch CT, Earn D 2004 Vaccination and the theory of games. Proc. Natl Acad. Sci. USA 101, 13 391–13 394. (10.1073/pnas.0403823101) PMC516577 15329411

[36] Bhattacharyya S, Bauch CT 2010 A game dynamic model for delayer strategies in vaccinating behaviour for pediatric infectious diseases. J. Theor. Biol. 267, 276–282. (10.1016/j.jtbi.2010.09.005) 20831873

[37] UNWTO. 2006 International tourist arrivals by country of destination—Europe. *Tourism Market Trends*, Annex 7.

[38] Lanzieri G 2006 Long-term population projections at national level. Eurostat: Stat. Focus, Popul. Soc. Conditions 3, 1–7.

[39] UNWTO. 2006 International tourist arrivals by country of destination—Africa. *Tourism Market Trends*, Annex 4.

[40] UN. 2010 World population prospects, the 2010 revision. See http://esa.un.org/unpd/wpp/Excel-Data/population.htm (accessed 1 March 2013).

[41] Barrett S 2005 The theory of international environmental agreements. In Handbook of environmental economics, vol. 3 (eds Maler KG, Vincent JR), pp. 1457–1516. Amsterdam, The Netherlands: Elsevier See http://www.sciencedirect.com/science/handbooks/15740099.

[42] Barrett S 2003 Increasing participation and compliance in international climate change agreements. Int. Environ. Agreements: Politics, Law Econ. 3, 349–376. (10.1023/B:INEA.0000005767.67689.28)

[43] Carraro C, Siniscalco D 1995 R&D cooperation and the stability of international environmental agreements. CEPR Discussion Papers, No.1154. London, UK: CEPR.

[44] Anderson RM, Fraser C, Ghani AC, Donnelly CA, Riley S, Ferguson NM, Leung GM, Lam TH, Hedley AJ 2004 Epidemiology, transmission dynamics and control of SARS: the 2002–2003 epidemic. Phil. Trans. R. Soc. Lond. B 359, 1091–1105. (10.1098/rstb.2004.1490) 15306395 PMC1693389

[45] Bogoch II et al. 2015 Assessment of the potential for international dissemination of Ebola virus via commercial air travel during the 2014 west African outbreak. Lancet 385, 29–35. (10.1016/S0140-6736(14)61828-6) 25458732 PMC4286618

[46] Gostin LO, Lucey D, Phelan A 2014 The Ebola epidemic: a global health emergency. JAMA 312, 1095–1096. (10.1001/jama.2014.11176) 25111044

[47] Hollingsworth TD, Ferguson NM, Anderson RM 2006 Will travel restrictions control the international spread of pandemic influenza? Nat. Med. 12, 497–499. (10.1038/nm0506-497) 16675989

[48] Poletto C et al. 2014 Assessing the impact of travel restrictions on international spread of the 2014 West African Ebola epidemic. Euro Surveill. 19, 20936 (10.2807/1560-7917.ES2014.19.42.20936) 25358040 PMC4415609

[49] Ferguson NM, Cummings DAT, Fraser C, Cajka JC, Cooley PC, Burke DS 2006 Strategies for mitigating an influenza pandemic. Nature 442, 448–452. (10.1038/nature04795) 16642006 PMC7095311

[50] Colizza V, Barrat A, Barthelemy M, Valleron A-J, Vespignani A 2007 Modeling the worldwide spread of pandemic influenza: baseline case and containment interventions. PLoS Med. 4, e13 (10.1371/journal.pmed.0040013) 17253899 PMC1779816

[51] Bajardi P, Poletto C, Ramasco JJ, Tizzoni M, Colizza V, Vespignani A 2011 Human mobility networks, travel restrictions, and the global spread of 2009 H1N1 pandemic. PLoS ONE 6, e16591 (10.1371/journal.pone.0016591) 21304943 PMC3031602

[52] Knobler S, Mahmoud A, Lemon S, Mack A, Sivitz L, Oberholtzer K, Institute of Medicine (US) Forum on Microbial Threats. 2004 Learning from SARS: preparing for the next disease outbreak: workshop summary. Washington, DC: National Academies Press (US). 22553895

[53] Beutels P, Jia N, Zhou Q-Y, Smith R, Cao W-C, de Vlas SJ 2009 The economic impact of SARS in Beijing, China. Trop. Med. Int. Health 14, 85–91. (10.1111/j.1365-3156.2008.02210.x) 19508435

[54] Epstein JM, Goedecke DM, Yu F, Morris RJ, Wagener DK, Bobashev GV 2007 Controlling pandemic flu: the value of international air travel restrictions. PLoS ONE 2, e401 (10.1371/journal.pone.0000401) 17476323 PMC1855004

[55] The World Bank 2015 United States data 2013. See data.worldbank.org. 1–4.

[56] Grout L et al. 2014 Local discrepancies in measles vaccination opportunities: results of population-based surveys in Sub-Saharan Africa. BMC Public Health 14, 193 (10.1186/1471-2458-14-193) 24559281 PMC3938072

[57] Metcalf CJE, Tatem A, Bjornstad ON, Lessler J, O'Reilly K, Takahashi S, Cutts F, Grenfell BT 2015 Transport networks and inequities in vaccination: remoteness shapes measles vaccine coverage and prospects for elimination across Africa. Epidemiol. Infect. 143, 1457–1466. (10.1017/S0950268814001988) 25119237 PMC4411642

[58] LaForce FM, Okwo-Bele J-M 2011 Eliminating epidemic group A meningococcal meningitis in Africa through a new vaccine. Health Aff. (Millwood) 30, 1049–1057. (10.1377/hlthaff.2011.0328) 21653956

[59] Trotter CL, Cibrelus L, Fernandez K, Lingani C, Ronveaux O, Stuart JM 2015 Response thresholds for epidemic meningitis in sub-Saharan Africa following the introduction of MenAfriVac^®^. Vaccine 33, 6212–6217. (10.1016/j.vaccine.2015.09.107) 26463444

[60] MenAfriCar consortium. 2015 The diversity of meningococcal carriage across the African meningitis belt and the impact of vaccination with a group a meningococcal conjugate vaccine. J Infect Dis 212, 1298–1307. (10.1093/infdis/jiv211) 25858956 PMC4577048

[61] Bjornstad O, Finkenstadt B, Grenfell B 2002 Dynamics of measles epidemics: estimating scaling of transmission rates using a time series SIR model. Ecol. Monogr. 72, 169–184. (10.2307/3100023)

