# Supplementary material for: Self-enforcing regional vaccination agreements
Source: J R Soc Interface. 2016 Jan;13(114):20150907. doi: 10.1098/rsif.2015.0907 (PMC4759795; doi:10.1098/rsif.2015.0907)
Supplement: Supporting Material for “Self-enforcing regional vaccination agreements” [file rsif20150907supp1.pdf]

## Supporting Information for "Self-enforcing regional vaccination agreements"

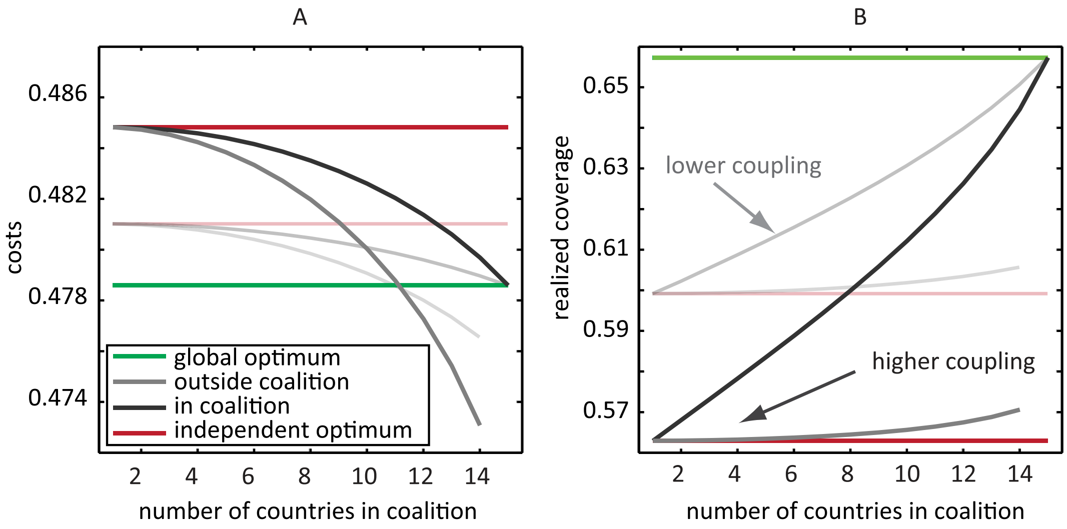


**Figure S1.** Effects of the strength of coupling on the (A) costs and (B) realized coverage for 15 coupled identical countries and increasing coalition size (x-axis). Green and red lines show global and independent optima, black and gray lines show respective optima for countries in coalition (signatories) and outside coalition (nonsignatories). Higher coupling ($\eta = 20\mu/(n\text{-}1)$) is indicated with opaque lines; transparent lines show lower coupling ($\eta=10\mu/(n\text{-}1)$). Other parameters: $R_0 = 5$, $a_i = 0.1$, $c_{Ii} = 5$.

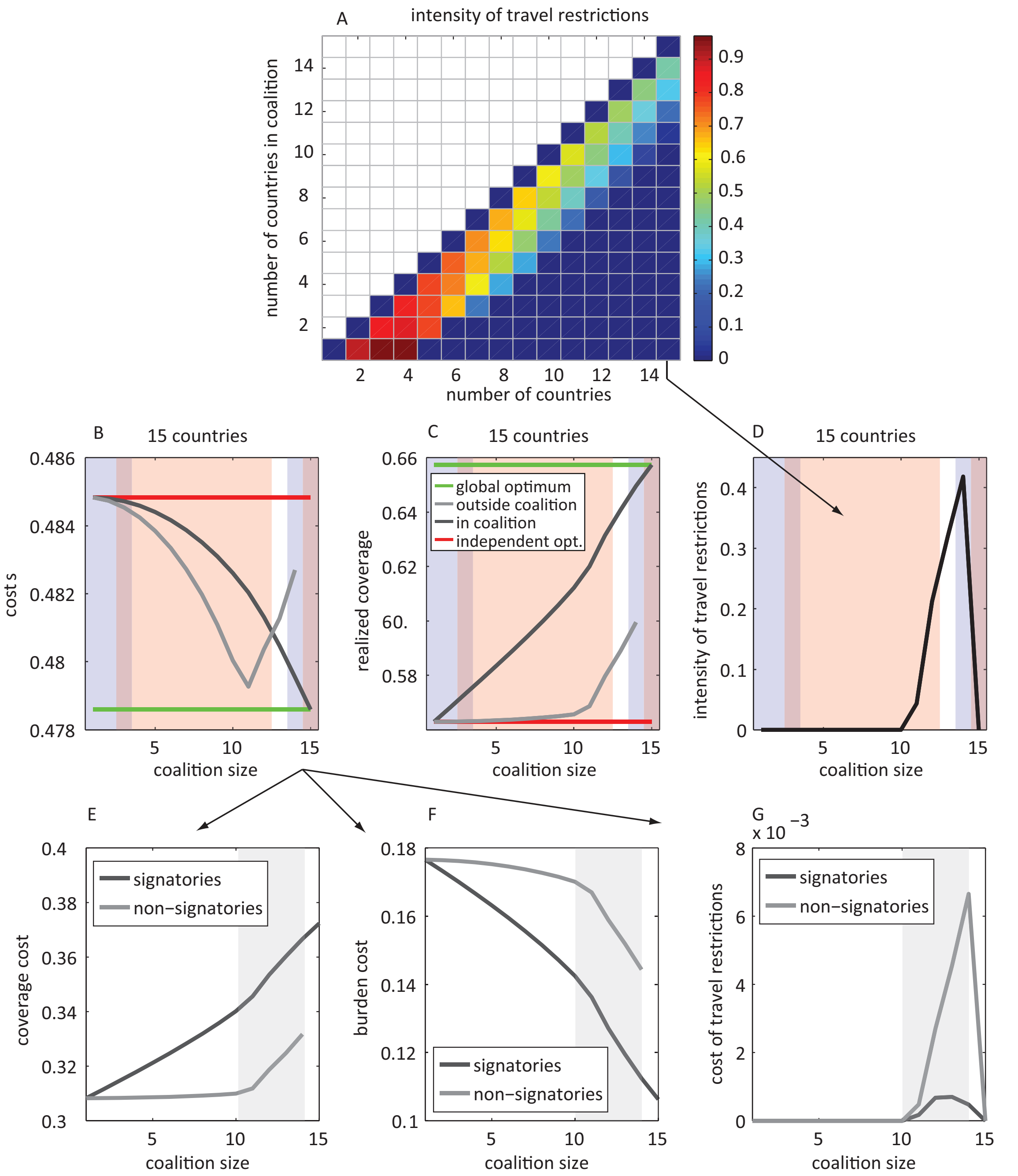


**Figure S2.** Multi-patch SIR model with travel restrictions. (A) The color shows the optimal intensity of travel restrictions imposed by signatories for increasing number of interconnected countries (x-axis) and increasing number of signatories (y-axis). Intensity of travel restrictions varies from no restrictions (dark blue) to complete isolation (dark red). B-G Details for 15 identical coupled countries. (B) Minimized total costs and optimal coverage (C) for countries inside the coalition (black line), outside of coalition (gray line), fully cooperative, global optimum (green line), and when countries are acting independently (red line). (D) Optimal travel restriction intensity. Blue shading in B-D shows internally stable coalitions, externally stable coalitions are shaded orange, and their overlap indicates self-enforcing coalitions. Total costs shown in B are a sum of costs of coverage (E), disease burden (F) and direct costs of travel restrictions (G). Shaded areas in E-F indicate coalitions that implement quarantine (positive quarantine level in D). $R_0$ = 5, (lower) coupling strength = $10\mu/(n\text{-}1)$, $a_i$=0.1, $c_{li}$ = 5. Implementing travel restrictions is assumed to be expensive, $q$ = 500.

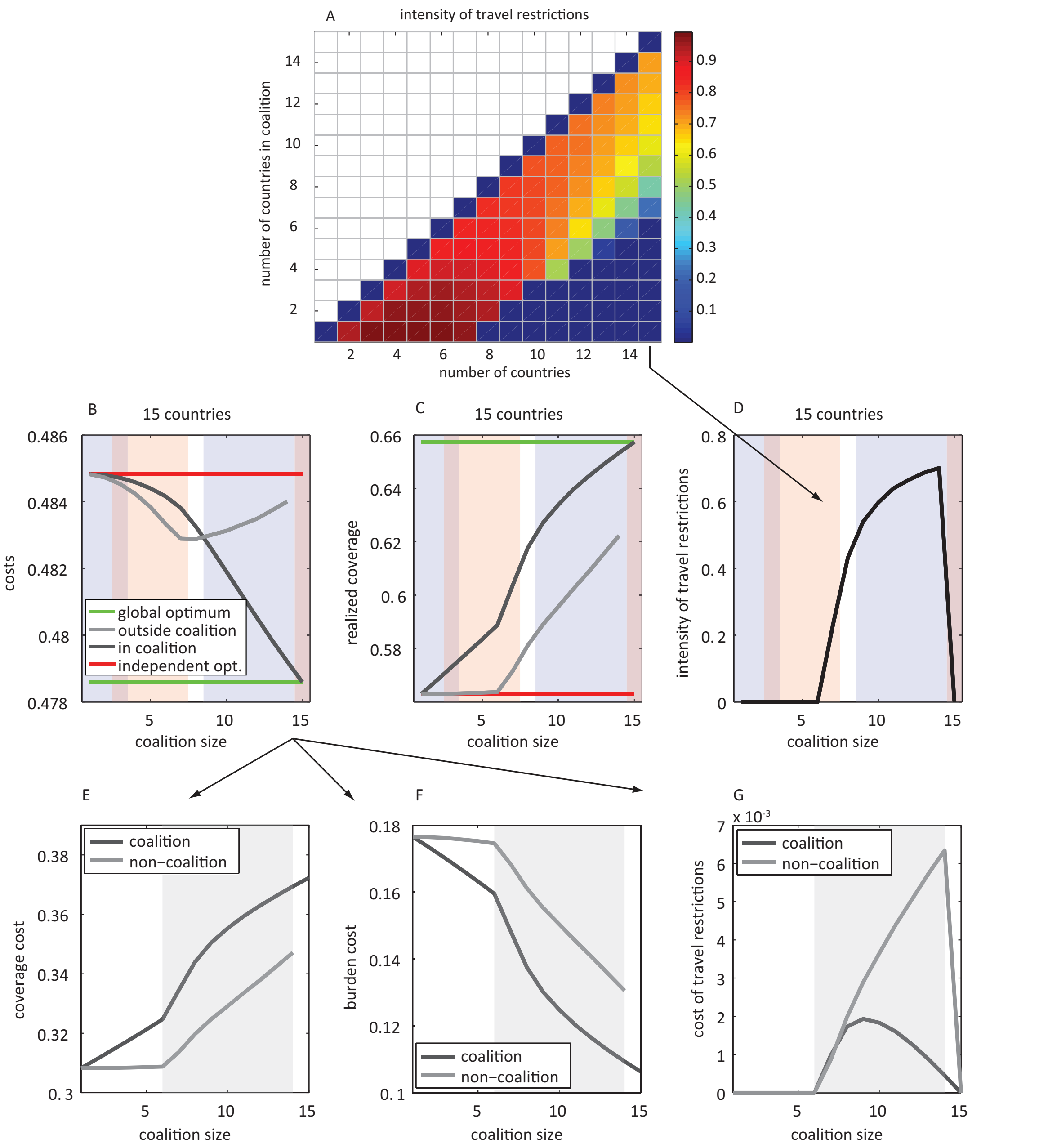


**Figure S3** Multi-patch SIR model with travel restrictions. (A) The color shows the optimal intensity of travel restrictions imposed by signatories for increasing number of interconnected countries (x-axis) and increasing number of signatories (y-axis). Intensity of travel restrictions varies from no restrictions (dark blue) to complete isolation (dark red). B-G Details for 15 identical coupled countries. (B) Minimized total costs and optimal coverage (C) for countries inside the coalition (black line), outside of coalition (gray line), fully cooperative, global optimum (green line), and when countries are acting independently (red line). (D) Optimal travel restriction intensity. Blue shading in B-D shows internally stable coalitions, externally stable coalitions are shaded orange, and their overlap indicates self-enforcing coalitions. Total costs shown in B are a sum of costs of coverage (E), disease burden (F) and direct costs of travel restrictions (G). Shaded areas in E-F indicate coalitions that implement quarantine (positive quarantine level in D). $R_0$ = 5, coupling strength = $20\mu/(n\text{-}1)$, $a_i$=0.1, $c_{Ii}$ = 5. Implementing travel restrictions is assumed to be inexpensive, $q$ = 1000 .

.

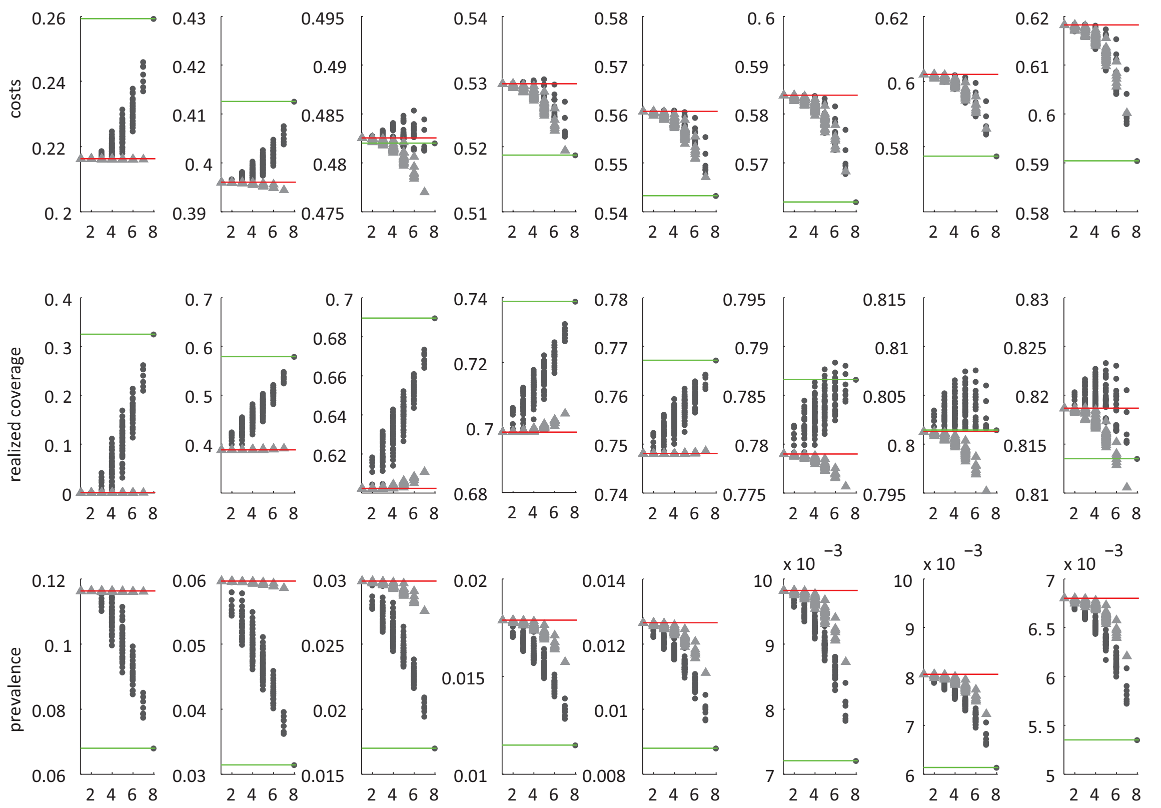


**Figure S4** Metapopulation of 8 countries where infection cost changes linearly from $c_1$ = 1 to $c_8$ = 15. $R_0$ = 5 for all countries, $a$ = 0.1 for all countries (details for Figure 4 in the main text). Columns organize results by country, with country 1 on the left, and country 8 on the right. Rows show resulting cost, realized coverage, and prevalence for each of the countries when in coalition (black circles) and when that country is not in the coalition (gray triangles). The x-axis of each plot shows the number of countries in the coalition; 1 – all countries act independently (local equilibrium shown in red), 8 – all countries are in the coalition (global optimum shown in green).

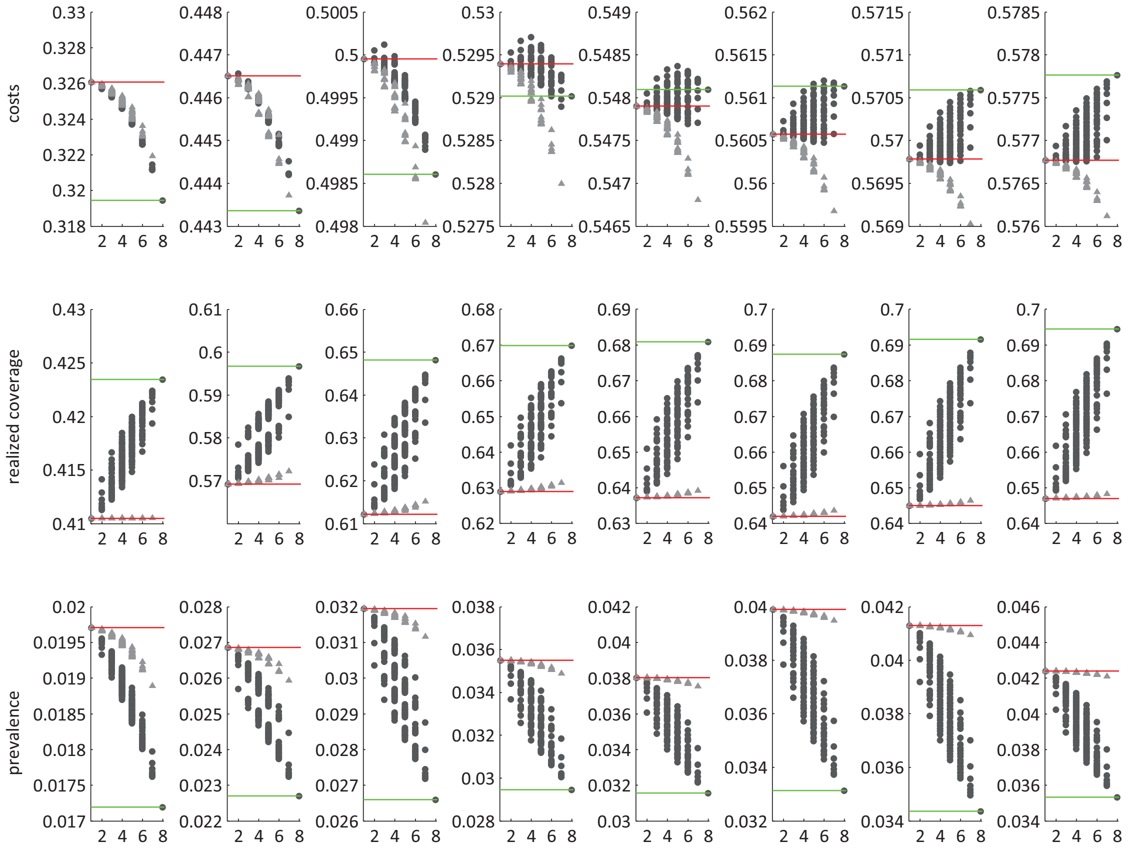


**Figure S5.** Metapopulation of 8 asymmetric countries where $R_0$ changes linearly from $R_{01}$ = 2 to $R_{08}$ = 15. $c$ = 5 for all countries, $a$ = 0.1 for all countries. Rows show resulting cost, realized coverage, and prevalence for each of the countries when in coalition (black circles) and when that country is not in the coalition (gray triangles). The x-axis of each plot shows the number of countries in the coalition; 1 – all countries act independently (local equilibrium shown in red), 8 – all countries are in the coalition (global optimum shown in green).

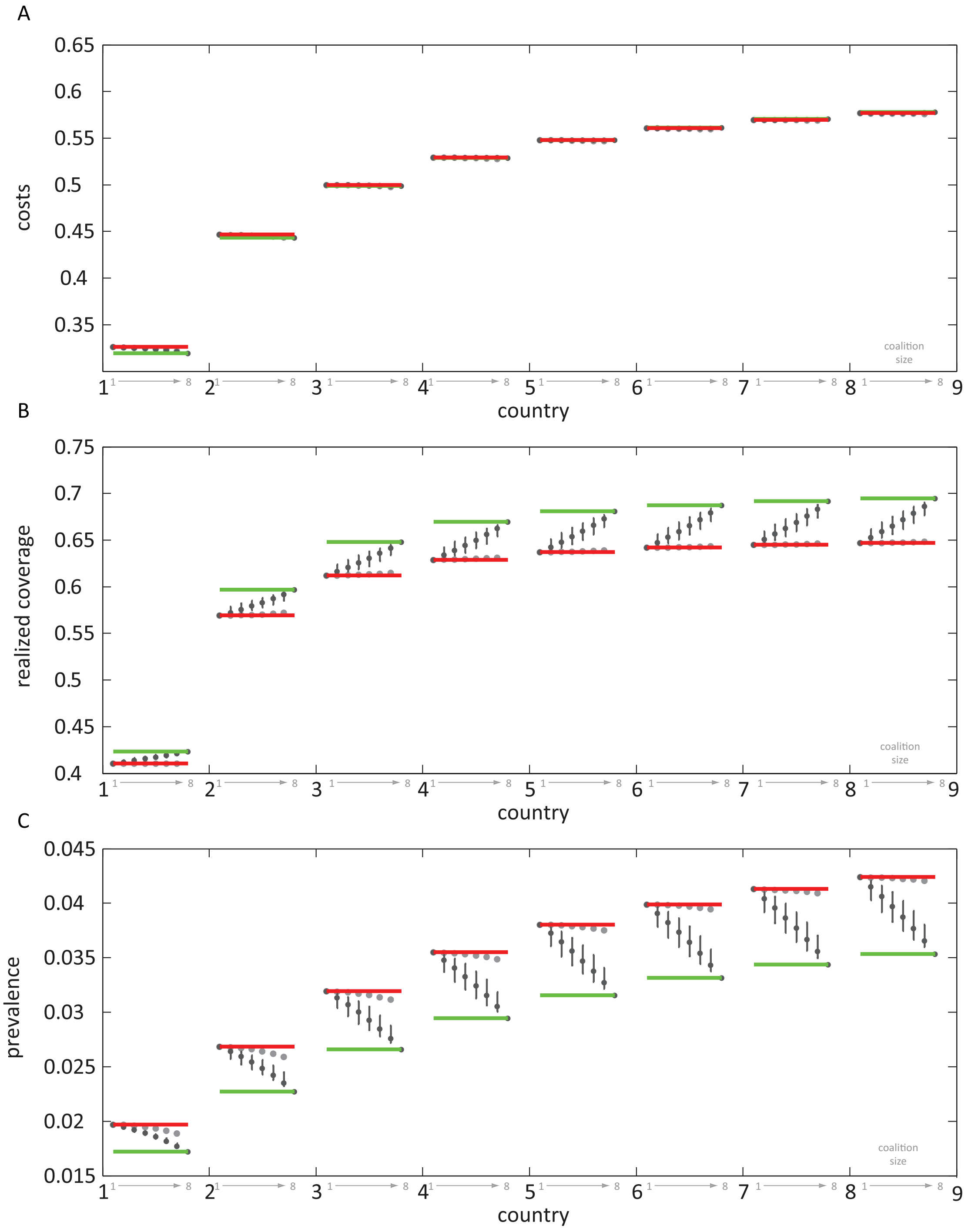


**Figure S6** Metapopulation of 8 asymmetric countries where $R_0$ changes linearly from $R_{01}$ = 2 to $R_{08}$ = 15. $c$ = 5 for all countries, $a$ = 0.1 for all countries (same as in Figure S5). Circles show mean costs (A), coverage (B) or prevalence (C) for each country and each coalition size when that country is in coalition (black) and outside of coalition (gray). For each country the coalition sizes are ordered from non-cooperative outcome to fully cooperative outcome. Whiskers show 5-th and 95-th quantiles of all possible coalitions for a particular coalition size and country. Red and green lines show independent and global optimum for each country, respectively.

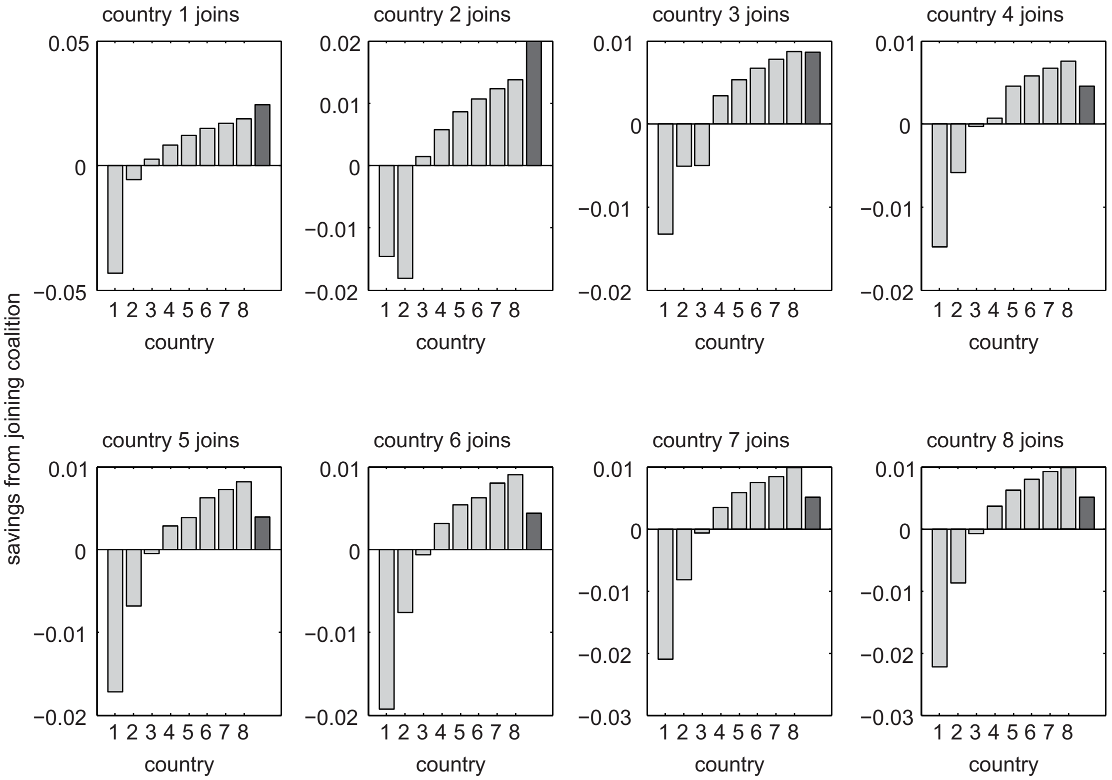


**Figure S7.** Savings (or costs, if negative) of joining a fully cooperative coalition of 8 asymmetric countries (all countries participate, $k = n$). Coalition increases in size from ($n$-1) to $n$. Savings per country are shown in gray and overall coalition savings are given in black. Cost of infection parameter varies linearly across countries from $c_{I1}$ = 1 for country 1, and $c_{I8}$ = 15 for country 8. $R_0$ = 5, coupling strength = $10\mu/(n\text{-}1)$, $a_i$=0.1.
